# Response to “Letter to Editor From Ishaq MU et al: ‘Semaglutide Concurrently Improves Vascular and Liver Indices in Patients With Type 2 Diabetes and Fatty Liver Disease’”

**DOI:** 10.1210/jendso/bvae218

**Published:** 2024-12-04

**Authors:** Emmanouil Korakas, Aikaterini Kountouri, George Pavlidis, Evangelos Oikonomou, Emmanouil Vrentzos, Eleni Michalopoulou, Vasiliki Tsigkou, Konstantinos Katogiannis, Loukia Pliouta, Konstantinos Balampanis, Sotirios Pililis, Konstantinos Malandris, Apostolos Tsapas, Gerasimos Siasos, Ignatios Ikonomidis, Vaia Lambadiari

**Affiliations:** 2nd Department of Internal Medicine Research Unit and Diabetes Centre Attikon Hospital Medical School, National and Kapodistrian University of Athens, 12462 Athens, Greece; 2nd Department of Internal Medicine Research Unit and Diabetes Centre Attikon Hospital Medical School, National and Kapodistrian University of Athens, 12462 Athens, Greece; 2nd Department of Cardiology Laboratory of Preventive Cardiology and Echocardiography Department Attikon Hospital Medical School, National and Kapodistrian University of Athens,12462 Athens, Greece; 3rd Department of Cardiology, National and Kapodistrian University of Athens, Medical School, Sotiria Chest Disease Hospital, 11527 Athens, Greece; Rheumatology and Clinical Immunology Unit, 4th Department of Internal Medicine, Attikon University Hospital, Joint Rheumatology Program, National and Kapodistrian University of Athens Medical School, 12462 Athens, Greece; 2nd Department of Cardiology Laboratory of Preventive Cardiology and Echocardiography Department Attikon Hospital Medical School, National and Kapodistrian University of Athens,12462 Athens, Greece; 3rd Department of Cardiology, National and Kapodistrian University of Athens, Medical School, Sotiria Chest Disease Hospital, 11527 Athens, Greece; 2nd Department of Cardiology Laboratory of Preventive Cardiology and Echocardiography Department Attikon Hospital Medical School, National and Kapodistrian University of Athens,12462 Athens, Greece; 2nd Department of Internal Medicine Research Unit and Diabetes Centre Attikon Hospital Medical School, National and Kapodistrian University of Athens, 12462 Athens, Greece; 2nd Department of Internal Medicine Research Unit and Diabetes Centre Attikon Hospital Medical School, National and Kapodistrian University of Athens, 12462 Athens, Greece; 2nd Department of Internal Medicine Research Unit and Diabetes Centre Attikon Hospital Medical School, National and Kapodistrian University of Athens, 12462 Athens, Greece; Clinical Research and Evidence-Based Medicine Unit, Aristotle University of Thessaloniki, 54124 Thessaloniki, Greece; Clinical Research and Evidence-Based Medicine Unit, Aristotle University of Thessaloniki, 54124 Thessaloniki, Greece; 3rd Department of Cardiology, National and Kapodistrian University of Athens, Medical School, Sotiria Chest Disease Hospital, 11527 Athens, Greece; 2nd Department of Cardiology Laboratory of Preventive Cardiology and Echocardiography Department Attikon Hospital Medical School, National and Kapodistrian University of Athens,12462 Athens, Greece; 2nd Department of Internal Medicine Research Unit and Diabetes Centre Attikon Hospital Medical School, National and Kapodistrian University of Athens, 12462 Athens, Greece

We greatly appreciate the comments made by Ishaq et al regarding our work [1]. Indeed, the previous studies mentioned by the authors are also cited in our article, and the results are comparable, highlighting the need for further, large-scale studies regarding the effect of different GLP-1RAs on liver steatosis and, eventually, fibrosis.

Regarding the study sample, we should emphasize that the primary outcome of our study was the determination of the changes of controlled attenuation parameter (CAP) score and, therefore, for the sample size calculation of the 2 study groups (treatment and control group), we performed power calculation a priori based on an initial pilot study. The pilot sample included 10 consecutive patients in each group (semaglutide [GLP-1RA] and control [dipeptidyl peptidase 4 inhibitors]). The percentage change of CAP score (ΔCAP%) at 4 months was measured for the purpose of the power analysis (semaglutide 10.8% and control 4%, respectively). To detect a relevant reduction in CAP score with α = 0.05, power = 0.80, and SD = 9.5, at least n = 24 patients in each group are necessary for the conduction of the study.

All patients in our study had type 2 diabetes mellitus and, therefore, were advised to maintain a low glycemic index diet and a decent level of physical activity, namely at least 150 minutes of moderate-intensity physical activity every week. Indeed, no structured dietary questionnaires were dispensed to the patients and their compliance with exercise modules was not reported, which could be considered limitations of our study. It must be noted, however, that this is not a common practice in other similar studies in the literature.

Liver biopsy remains the “golden standard” for diagnosis and staging of metabolic dysfunction-associated steatotic liver disease (MASLD) [2]. However, the risk of complications associated with the procedure renders it neither a cost-effective nor a practical method for large-scale implementation. According to the most recent European Association for the Study of the Liver-European Association for the Study of Diabetes-European Association for the Study of Obesity Clinical Practice Guidelines [3], FIB-4 is the first step in noninvasive assessment of the risk for advanced fibrosis and liver-related outcomes in patients with type 2 diabetes mellitus and, according to its value, a further evaluation with FibroScan might be suggested. The Fatty Liver Index is an index score with sensitivity and specificity at 76% and 87%, respectively, but despite its reproducibility, its inherent low ability to distinguish the severity of steatosis limits its widespread use in clinical practice [4]. The same applies for Hepatic Steatosis Index, which has a high specificity (92%) for detecting nonalcoholic fatty liver disease, but low sensitivity (46%) [5]. The AST-to-platelet Ratio Index can effectively differentiate between patients with advanced fibrosis and no fibrosis, but its utility in intermediate stages is rather limited due to poor sensitivity [6]. In short, no noninvasive technique seems to stand out for all patients with the highly heterogenic entity of MASLD, and the validity of all scores should be tested against the newly defined criteria of the disease.

## Clinical Trial Information

ClinicalTrials.gov ID: NCT06301191
